# The transcription factor *PnMYB38* orchestrates methyl jasmonate-induced saponin biosynthesis in *Panax notoginseng*

**DOI:** 10.1093/hr/uhag052

**Published:** 2026-02-18

**Authors:** Zhi Yang, Lifang Yang, Zhiyao Zhu, Min Li, Jiae Hou, Shuying Wang, Huanzhen Wu, Qian Yang, Xiuming Cui, Yonghong Tao, Ye Yang, Yuan Liu

**Affiliations:** Faculty of Life Science and Technology, Kunming University of Science and Technology, Kunming, Yunnan 650500, China; Faculty of Life Science and Technology, Kunming University of Science and Technology, Kunming, Yunnan 650500, China; Faculty of Life Science and Technology, Kunming University of Science and Technology, Kunming, Yunnan 650500, China; Faculty of Life Science and Technology, Kunming University of Science and Technology, Kunming, Yunnan 650500, China; Faculty of Life Science and Technology, Kunming University of Science and Technology, Kunming, Yunnan 650500, China; Faculty of Life Science and Technology, Kunming University of Science and Technology, Kunming, Yunnan 650500, China; Faculty of Life Science and Technology, Kunming University of Science and Technology, Kunming, Yunnan 650500, China; Faculty of Life Science and Technology, Kunming University of Science and Technology, Kunming, Yunnan 650500, China; Yunnan Provincial Key Laboratory of Panax notoginseng, Kunming 650500, China; Key Laboratory of Panax notoginseng Resources Sustainable Development and Utilization of State Administration of Traditional Chinese Medicine, Kunming 650500, China; Kunming Key Laboratory of Sustainable Development and Utilization of Famous-Region Drug, Kunming 650500, China; Faculty of Life Science and Technology, Kunming University of Science and Technology, Kunming, Yunnan 650500, China; Yunnan Provincial Key Laboratory of Panax notoginseng, Kunming 650500, China; Key Laboratory of Panax notoginseng Resources Sustainable Development and Utilization of State Administration of Traditional Chinese Medicine, Kunming 650500, China; Kunming Key Laboratory of Sustainable Development and Utilization of Famous-Region Drug, Kunming 650500, China; Wenshan Academy of Agricultural Sciences, Wenshan, Yunnan 663000, China; Faculty of Life Science and Technology, Kunming University of Science and Technology, Kunming, Yunnan 650500, China; Yunnan Provincial Key Laboratory of Panax notoginseng, Kunming 650500, China; Key Laboratory of Panax notoginseng Resources Sustainable Development and Utilization of State Administration of Traditional Chinese Medicine, Kunming 650500, China; Kunming Key Laboratory of Sustainable Development and Utilization of Famous-Region Drug, Kunming 650500, China; Faculty of Life Science and Technology, Kunming University of Science and Technology, Kunming, Yunnan 650500, China; Yunnan Provincial Key Laboratory of Panax notoginseng, Kunming 650500, China; Key Laboratory of Panax notoginseng Resources Sustainable Development and Utilization of State Administration of Traditional Chinese Medicine, Kunming 650500, China; Kunming Key Laboratory of Sustainable Development and Utilization of Famous-Region Drug, Kunming 650500, China

## Abstract

*Panax notoginseng* is an important medicinal plant, and saponins are the primary active components that are the key determinants of pharmaceutical quality. Research has indicated that methyl jasmonate (MeJA) enhances the saponins accumulation in *P. notoginseng*, but the specific MeJA-responsive transcription factors (TFs) that regulate this process remains unidentified. Given the critical role of *MYB* TFs in the regulation of plant secondary metabolism, this study aimed to elucidate the regulatory mechanisms of the *MYB* TF family in *P. notoginseng* under MeJA treatment. Genome-wide screening led to the identification 110 *MYB* genes, followed by a comprehensive analysis of their phylogenetic relationships, conserved motifs, gene structures, cis-acting elements, chromosomal localization, and collinearity. Integrated transcriptomic and metabolomic analyses showed that MeJA treatment significantly altered the expression patterns of 84 *MYB* genes while also promoting saponin accumulation in *P. notoginseng* leaves. Co-expression network analysis revealed a significant correlation between *PnMYB38* and saponin metabolites, highlighting the pivotal regulatory function of this TF. Subcellular localization experiments confirmed nuclear localization of *PnMYB38*. Yeast one-hybrid, electrophoretic mobility shift assay, and dual-luciferase assays demonstrated that *PnMYB38* directly and specifically bound to the promoters of key saponin biosynthesis genes (*PnSE* and *PnDS*), thereby inducing their expression. This study comprehensively characterized the functional role of *PnMYB38* in regulating MeJA-mediated saponin biosynthesis in *P. notoginseng*, proposed a ‘MeJA-*PnMYB38*-saponin biosynthesis’ regulatory network that provided novel insights into the transcriptional regulatory mechanism of saponin biosynthesis, and established a foundation for molecular breeding targeting *MYB* TFs and metabolic engineering.

## Introduction

*Panax notoginseng*, a valuable medicinal plant within the genus *Panax*, has garnered considerable attention due to its diverse array of bioactive compounds. Studies have showed that it produces a wide range of secondary metabolites, including saponins, flavonoids, polysaccharides, alkaloids, volatile oils, and sterols. Saponins are the principal active components, exhibiting diverse pharmacological properties including anti-inflammatory, antitumor, cardioprotective, antifibrotic, antiaging, and antioxidant effects [1, 2]. Therefore, investigating the transcriptional regulatory mechanisms of saponins biosynthesis in *P. notoginseng* is of considerable significance for both elucidating the molecular basis and guiding metabolic engineering.

Saponins biosynthesis is initiated from the mevalonic acid and methylerythritol phosphate pathways [3]. The pathway involves key enzymes including farnesyl pyrophosphate synthase (FPS), squalene synthase (SS), squalene epoxidase (SE), oxidosqualene cyclase (OSC), and the ginsenoside-specific dammarenediol-II synthase (DS), which collectively construct the dammarane-type triterpenoid skeleton [4, 5]. The final structural diversity of ginsenosides is achieved through subsequent oxidative modification by cytochrome P450 enzymes (CYP450s) and glycosylation by glycosyltransferases (GTs) [6–8].

The transcriptional regulatory network of ginsenoside biosynthesis is multilayered and is mediated by transcription factors (TFs) from families such as *MYB*, *bHLH*, *WRKY*, and *NAC* [9–14]. Among these, *MYB* TFs represent one of the largest TF families in plants and are characterized by a conserved N-terminal DNA-binding domain (the *MYB* domain), which typically comprising one to four imperfect repeats (R1, R2, R3, and R4). *MYB* family play crucial roles in plant growth, development, metabolic regulation, and responses to biotic and abiotic stresses [15]. They are recognized as key regulators of secondary metabolic pathways that control the biosynthesis of saponins, flavonoids, anthocyanins, and lignin [16–21]. Previous studies have identified *MYB* TFs such as *PnMYB1*, *PnMYB*2, and *PnMYB*4 through homologous cloning and demonstrated their direct regulation of saponin biosynthetic genes. *PnMYB1* upregulates the expression of *PnFPS*, *PnSS*, and *PnDS*, thereby promoting the accumulation of saponins (notoginsenoside R_1_, ginsenoside Rg_1_, and ginsenoside Re) [17]. *PnMYB2* enhances the expression of *PnSS* and *PnSE1* by binding to their promoters [19], and silencing *PnMYB4* increases saponin content by alleviating its repressive effect on key biosynthetic enzyme genes [17].

The expression of many TF genes is often modulated by environmental cues and phytohormone signaling [22]. Phytohormones, including methyl jasmonate (MeJA), abscisic acid, naphthalene acetic acid, and salicylic acid, are also essential regulators of secondary metabolites [23]. Among these, jasmonic acid (JA) and its derivative, MeJA, are prominent. MeJA markedly enhanced the saponin content in *P. notoginseng* callus tissue, correlating with the upregulation of key enzyme genes (e.g. *PnSS*, *PnSE*, *PnDS*, *PnCYP*, *PnUGT*) [24], and induced the accumulation of protopanaxadiol (PPD)-type saponins in *P. notoginseng* leaves [25]. The *MYB* TF family is a key responder in the JA signaling pathway, capable of directly perceiving JA signals to regulate downstream gene expression and influence secondary metabolite accumulation [22]. For example, *PgMYB2* enhances saponin synthesis in response to MeJA induction in *P. ginseng* [26] and *AbMYB11* enhanced disease resistance in *Agaricus bisporus* in response to MeJA induction [27]. MeJA enhances saponin accumulation in *Platycodon grandiflorus* by upregulating the TF *PgbHLH28*, which in turn activates key biosynthetic genes (*PgHMGR2* and *PgDXS2*) in the saponin pathway [28]. *SmMYB39*-*SmMBW* module mediates JA signaling and synergistically regulates the biosynthesis of phenolics and diterpenoids in *Salvia miltiorrhiza* [29].

Although both MeJA and *MYB* TFs are known to play important roles in saponin synthesis in *P. notoginseng*, a systematic understanding of how phytohormones signals regulate saponin biosynthesis through *MYB* networks remains unclear. To address the lack of a systematic understanding of the ‘MeJA-*MYB*-saponin’ regulatory module, this study performed a genome-wide analysis of the *MYB* family in *P. notoginseng*, integrated with multi-omics profiling. We identified *PnMYB38* as a central MeJA-responsive regulator. Functional validation further confirmed that *PnMYB38* directly activates key biosynthetic genes (*PnSE* and *PnDS*), thereby elucidating a well-defined ‘MeJA-*PnMYB38*-saponin biosynthesis’ transcriptional module. Collectively, these findings provide novel mechanistic insights into hormone-mediated saponin biosynthesis and identify a promising target for metabolic engineering.

## Results

### Identification and characterization of the *MYB* gene family members

Candidate *MYB* family members were identified using the HMM and BLAST. The presence of complete *MYB* domains was further verified using SMART and NCBI-CDD online tools, resulting in the selection of 110 family members (Table S1). These included 107 R2R3-MYB genes and 4 R1R2R3-MYB genes, classified based on the number of R repeats they contained. These genes were named *PnMYB1* to *PnMYB107* and *PnMYB3R1* to *PnMYB3R4*. The MYB protein lengths vary from 79 amino acids (*PnMYB82*) to 1021 amino acids (*PnMYB3R4*), with molecular weights ranging from 9.23 kDa (*PnMYB82*) to 112.95 kDa (*PnMYB3R4*). The theoretical isoelectric points (pI) range from 4.59 (*PnMYB26*) to 10.83 (*PnMYB46*), with majority having a pI slightly above 7.0, indicating that more than half of the proteins are rich in basic amino acids (Table S2). Physicochemical analyses suggested that these proteins are hydrophilic, thermally stable, and enriched in basic amino acids. Protein localization predictions indicated that most *PnMYB* TFs (>95%) were localized in the nucleus.

**Figure 1:**
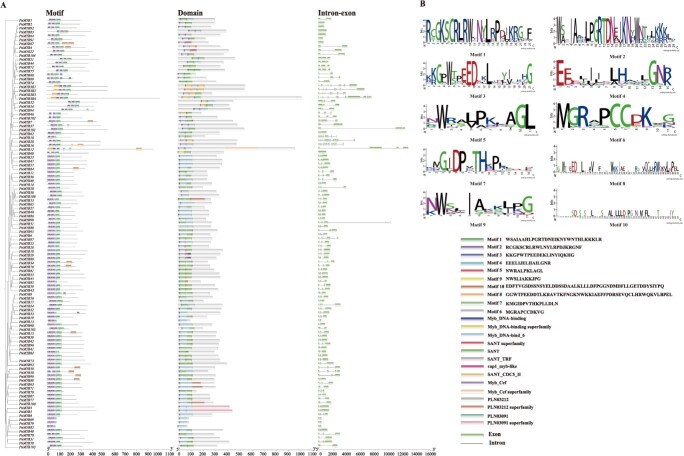
Gene structure, conserved motif, and conserved domain analysis of *PnMYB* genes. (A) *PnMYB* genes family phylogenetic tree and conserved motif, domains of the *PnMYB* genes family, exon–intron regions of the *PnMYB* genes family. (B) The 10 motifs of *PnMYB* genes in detail.

To characterize MYB proteins, conserved motifs, domains, and gene structure analyses were performed using the MEME suite and TBtools. Conserved motifs frequently corresponding to protein-binding sites essential for biological processes were characterized in 110 *PnMYB* proteins. Ten such motifs (11–50 amino acids in length) were identified, which exhibited substantial variation in both size and amino acid conservation. Nearly all R2R3-MYB family members contain three highly conserved motifs (motif1, motif2, and motif3) arranged in the order 3-2-1. Exceptions were observed *PnMYB70, PnMYB80*, and *PnMYB83* lack motif1. Additionally, *PnMYB10*, *PnMYB12, PnMYB34, PnMYB56, PnMYB70, PnMYB80, PnMYB83*, and *PnMYB86* lack motif2, while *PnMYB95* did motif3 (Fig. 1A). To further interpret the motif diversity, the 10 predicted motifs were annotated using WebLogo (Fig. 1B). These results suggest that the *MYB* genes have undergone evolutionary divergence, with motifs serving as potential markers for gene identification. Phylogenetic analysis revealed that members of the same subgroup shared similar motif compositions and arrangements, whereas distinct subgroups exhibited notable differences, implying functional divergence among the genes.

The conserved structural domains within the same phylogenetic branch were similar, and all genes contained MYB-related conserved domains such as Myb-DNA-binding and SANT (Fig. 1A; Table S3). Most of these domains were located at the N-terminus, whereas the rap1_myb-like domain was present only in *PnMYB22* and *PnMYB67*. Rap1 (repressor-activator protein) is a key DNA-binding protein in *Saccharomyces cerevisiae* that regulates gene expression by functioning in gene activation and repression [30]. Among the 71 *PnMYBs*, 5 contained Myb-DNA-binding and SANT-related domains, 24 contained 6 such domains, 17 contained 7, and *PnMYB12* contained 9 Myb-DNA-binding and SANT-related domains.

The number of exons in *PnMYBs* ranged from 1 to 12, and introns ranged from 0 to 11. Among these, 71 *PnMYB* genes contained two introns, accounting for the largest proportion (64.54%). Five *PnMYBs* contained three introns, while *PnMYB2*, *PnMYB60*, *PnMYB64*, *PnMYB67*, *PnMYB83*, *PnMYB91*, and *PnMYB92* lacked these introns (Fig. 1A). This suggests that intron gain in *P. notoginseng MYBs* may contribute to functional diversification, potentially enabling the evolution of additional biological roles.

### Cis-acting element analysis

**Figure 2:**
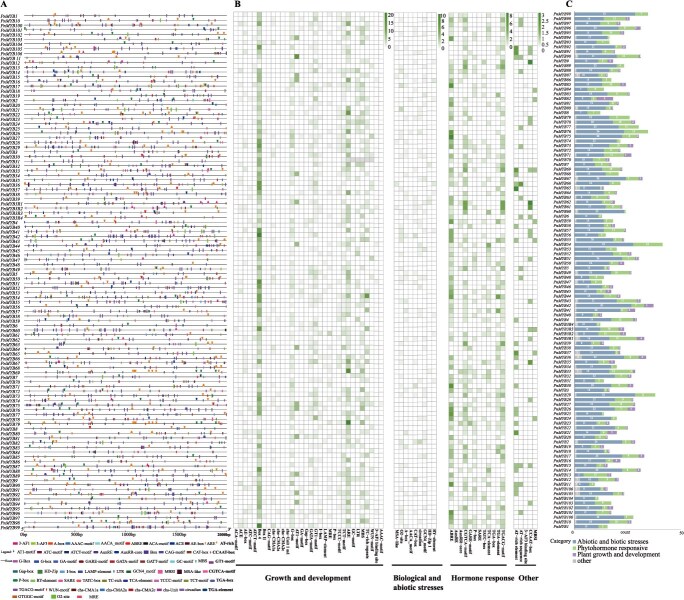
Analysis of *PnMYB* genes promoter elements of *P. notoginseng*. (A) Distribution pattern of cis-acting elements in the *PnMYB* promoter region. (B) Promoter region cis-acting element quantity heat map. (C) Number of *PnMYB* genes in growth and development, biological and abiotic, hormone response, and other.

Cis-acting elements within the promoter regions of the 110 *PnMYBs* were analysed using the PlantCARE database. The upstream sequences (2.0 kb) were examined (Fig. 2A; Table S4). The elements were categorized into four groups: biotic/abiotic stress response, plant growth and development, hormone response, and others (Fig. 2B and C; Tables S3, S4). The promoters of *PnMYB* genes harbour two core cis-regulatory element categories: biological and abiotic stress elements and hormone-responsive elements. High-frequency motifs included the TGACG motif (77 genes), CGTCA motif (77 genes), ARE (82 genes), ABRE (88 genes), G-box (86 genes), and Box 4 (106 genes). Biological and abiotic stress elements (31 types) included ARE (anaerobic induction; 82 genes), LTR (low-temperature response; 28 genes), TC-rich repeats (defense regulation; 39 genes), and the WUN-motif (wound stress response; 7 genes).

The growth and development elements encompass the O2-site (zein metabolism; 35 genes), CAT box (meristem expression; 24 genes), circadian elements (diurnal rhythm; 23 genes), HD-Zip 1 (mesophyll differentiation; 4 genes), GCN4-motif (endosperm expression; 27 genes), AACA motif (endosperm development suppression; 2 genes), RY element (seed specificity; 10 genes), and MSA-like elements (cell cycle control; 5 genes). The hormone-responsive elements included CGTCA/TGACG motifs (MeJA response; 77 genes), ABRE (ABA signalling; 88 genes), P-box/GARE-motif (gibberellin response; 39/19 genes), TCA-element (salicylic acid signalling; 43 genes), and auxin-related elements (AuxRR-core, AuxRE, TGA-element, and TGA-box). Three TF-binding elements were identified: MBS (MYB-binding site involved in drought induction; 54 genes), MRE (light-responsive MYB-binding site; 28 genes), and MBSI (MYB-binding site involved in flavonoid biosynthesis; 8 genes) (Table S5). These results imply that *PnMYB* genes are likely involved in the growth, development, and stress responses of *P. notoginseng*.

### Chromosomal distribution, phylogeny and collinearity analysis of *PnMYB* genes

**Figure 3:**
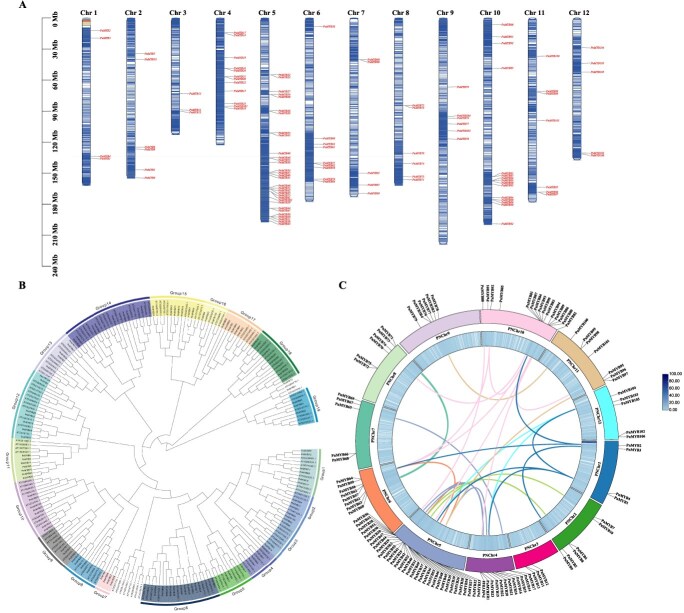
Chromosomal localization, phylogenetic relationship, and the collinearity analysis within the genome of *PnMYBs*. (A) Chromosomal localization of *P. notoginseng* MYB genes. (B) A maximum likelihood phylogenetic tree of *PnMYB* genes and *AtMYB* genes. The phylogenetic tree was constructed using the Maximum Likelihood (ML) method in IQ-TREE v2.2.6. Use the online site Evolview for evolutionary tree beautification. And the 19 major groups are marked with different color backgrounds. (C) The collinearity analysis within the genome of *PnMYB*.

The chromosomal localization of 110 *PnMYB* genes was identified through whole-genome database screening of *P. notoginseng*. Among these, 103 genes were unevenly distributed across the 12 chromosomes. Seven genes remained unmapped owing to incomplete genome sequencing. Chromosome 5 harboured the most genes (32), followed by chromosome 10 (15), chromosome 4 (12), and chromosome 6 (9); chromosomes 2, 8, 9, and 11 each contained 6 genes, chromosomes 7 and 12 had 5 each, chromosome 1 had 4, and chromosome 3 had the fewest (3) (Fig. 3A; Table S6).

To obtain comprehensive evolutionary information, full-length MYB protein sequences were aligned using ClustalW in MEGA11. A phylogenetic tree was constructed using IQ-TREE v2.2.6, using the maximum likelihood method, and incorporating *P. notoginseng* (110 sequences) and *Arabidopsis thaliana* (124 sequences). Based on the clustering patterns of MYBs in the evolutionary tree, the conserved domains of MYBs, and their distinct amino acid sequence motifs [31, 32], we classified MYB proteins into 19 subgroups. Among these, 18 groups contained *A. thaliana* R2R3-MYB genes, whereas group 7 did not include any *AtMYBs* and consisted of only four *PnMYBs*. This specific subfamily of *PnMYBs* may have evolutionarily diverged from those of other species (Fig. 3B; Table S7). Groups 6 and 14 contained the most MYB members (12 and 10, respectively), followed by groups 10, 11, 12, 15, and 18, whereas group 5, 16, and 19 had the fewest members with only three, respectively.

Syntenic blocks, indicative of the conserved gene order resulting from genome duplication and divergence, were analysed. Gene duplication plays a key role in family expansion and functional evolution [33] primarily through tandem and segmental duplications [34]. Synteny, GC content, and gene density were visualized in a Circos plot using the TBtools (Fig. 3C). Coloured lines connect syntenic gene pairs across chromosomes, with the inner blue ring representing gene density.

Within the *P. notoginseng* genome, 28 segmental and four tandem duplication events were identified among the 110 *PnMYB* genes (Table S8). Tandem duplicates included the following four gene pairs: *PnMYB32*/*PnMYB33*, *PnMYB41*/*PnMYB55*, *PnMYB66*/*PnMYB68*, and *PnMYB72*/*PnMYB75*. Segmental duplications were predominantly located on chromosome 5. The abundance of duplication events suggests that gene duplication, particularly segmental duplication, is a major driver of the expansion and diversification of the *PnMYB* gene family.

### MeJA treatment-induced significant transcriptome changes and qRT-PCR analysis of *PnMYB* genes

To elucidate the molecular mechanisms governing the metabolic response of *P. notoginseng MYB* TFs to MeJA induction, we performed transcriptome sequencing of *P. notoginseng* leaves to systematically examine the dynamic expression patterns of genes under MeJA treatment. RNA sequencing was performed using an Illumina HiSeq platform, which yielded ~10 Gb of high-quality reads per sample. PCA analysis revealed that PC1 and PC2 accounted for 27.04% and 24.85% of the total variance, respectively (Fig. S1). Samples from the control group (0 h) clustered tightly, whereas samples from all MeJA-treated time points (3, 6, and 24 h) were clearly separated from the control group along PC2, with the 24 h samples showing the most pronounced separation. Volcano plots display the differentially expressed genes (DEGs) identified by comparing each MeJA-treated group (3, 6, and 24 h posttreatment) with the untreated control (0 h) (Table S9). In each comparison, genes with |log2FC| > 1 and FDR < 0.05 were considered significant. In total, 1288 significant DEGs were consistently at all three time points, including 392 upregulated (red) and 896 downregulated (blue) genes (Fig. S2), indicating a rapid and sustained transcriptional response to MeJA in *P. notoginseng.*

**Figure 4:**
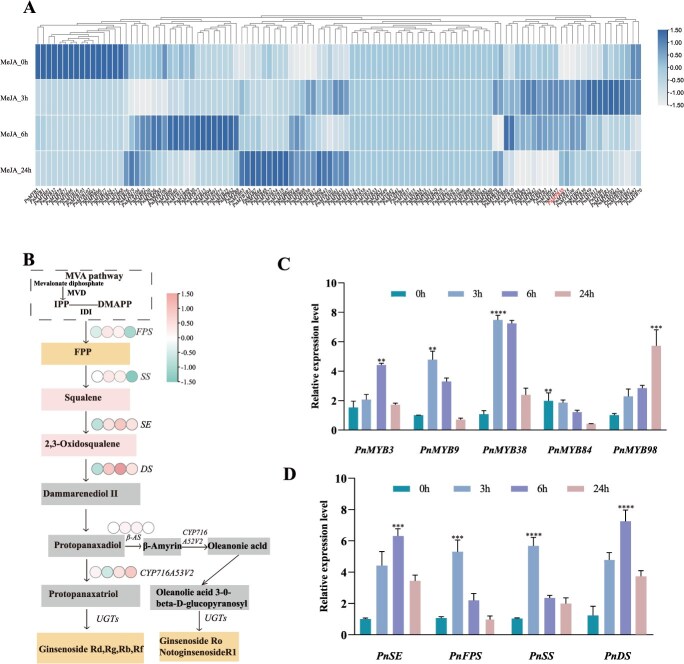
Expression analysis of *PnMYB* and saponin biosynthetic genes in response to MeJA treatment. (A) Heatmap of the differentially expressed 110 *PnMYB* genes family during MeJA treatment. (B) Heatmap analysis of biosynthetic gene expression dynamics in the saponin pathway of *P. notoginseng*. (C, D) RT-qPCR validation of MYB TF and key triterpenoid biosynthesis gene expression during MeJA treatment.

Among the 110 identified *MYB* genes, 84 exhibited significant dynamic expression at 0, 3, 6, and 24 h after MeJA treatment, whereas 26 showed no change. Temporal expression profiling revealed that 22, 25, and 17 *PnMYB* genes were significantly upregulated at 3, 6, and 24 h, respectively, whereas 41, 15, and 40 genes were downregulated at the same time points. At 3 h, the most highly expressed genes were *PnMYB33*, *PnMYB3R4*, *PnMYB13*, *PnMYB38*, *PnMYB101*, *PnMYB97*, *PnMYB82*, *PnMYB77*, *PnMYB14*, *PnMYB70*, PnM*YB3R1*, and *PnMYB59*. After 6 h, the most highly expressed genes were *PnMYB106*, *PnMYB30*, *PnMYB94*, *PnMYB25*, *PnMYB15*, *PnMYB96*, and *PnMYB*8. After 24 h, highly expressed genes included *PnMYB54*, *PnMYB44*, *PnMYB48*, *PnMYB88*, *PnMYB46*, *PnMYB18*, and *PnMYB4* (Fig. 4A).

This study analysed the dynamic expression profiles of key DEGs involved in the biosynthetic pathways of notoginsenosides in response to MeJA treatment. The results demonstrated that *PnSS* and *PnFPS* exhibited significant upregulation at 3 h post-treatment, while *PnSE* and *PnDS* showed sustained significant upregulation at 6 and 24 h. *PnCYP71653V2* (cytochrome P450) displayed an upward trend at 6 and 24 h, whereas β-AS (β-amyrin synthase) revealed no significant changes in expression at any time point (Fig. 4B). Based on the transcriptome data, we selected six differentially expressed *MYB* genes (*PnMYB3*, *PnMYB9*, *PnMYB38*, *PnMYB49*, *PnMYB84*, *PnMYB98*) and four saponin biosynthesis genes (*PnSS, PnSE, PnFPS, PnDS*) for qRT-qPCR validation. *PnMYB49* showed no significant expression, possibly due to its low abundance and tissue specificity. The other five *MYB* genes exhibited dynamic changes; *PnMYB3*, *PnMYB9*, *PnMYB38*, and *PnMYB98* were upregulated at 3 h and then declined, while *PnMYB84* decreased throughout. *PnMYB38* showed the most pronounced upregulation, suggesting a key role in the MeJA response (Fig. 4C). The expression trends of *PnMYB3*, *PnMYB9*, *PnMYB38*, and *PnMYB98* correlated well with the RNA-seq data, supporting the reliability of the data. Among the saponin pathway genes, *PnSS* and *PnFPS* were upregulated at 3 h and then decreased, whereas *PnSE* and *PnDS* were upregulated at both 3 and 6 h and declined only at 24 h (Fig. 4D). These results indicated the time-dependent regulation of saponin biosynthesis by MeJA.

### Metabolomic analysis

**Figure 5:**
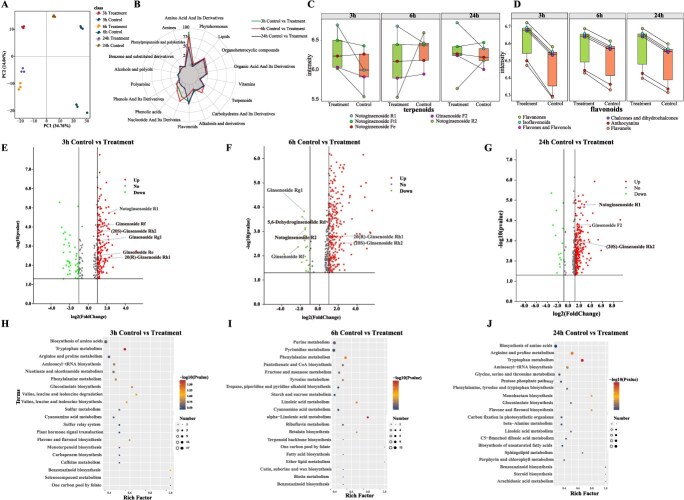
Evaluation of the influence of protoplasting genes on cell clustering. (A) PCA of metabolites: treatment vs. control at three time points. (B) Radar chart of metabolite proportions between treatment and control groups at three time points. (C, D) Differential analysis of terpenoids and flavonoids in treatment vs. control groups at three time points. (E–G) Volcano plots of DAMs between MeJA-treated and control groups at three time points. (H–J) KEGG enrichment plot of DAMs between MeJA-treated and control groups at three time points.

Principal component analysis (PCA) of the metabolites revealed a statistically significant separation between MeJA_3h and control_3h, MeJA_6h and control_6h, and MeJA_24h and control_24h. PC1 accounted for 34.76% of the variance, whereas PC2 accounted for 14.04%. The PCA results were further supported by a cluster analysis, which confirmed the sample grouping (Fig. 5A). Across all samples, 1212 metabolites were identified (Table S10), including 231 amino acids and their derivatives, 116 lipids, 109 flavonoids, 82 organic acids, 80 nucleotides and their derivatives, 64 terpenoids, 44 phenolic acids, 14 amines, 28 phenylpropanoids and polyketides, 38 alkaloids and their derivatives, 63 sugars and their derivatives, 28 phenols and their derivatives, 25 plant hormones, 16 vitamins, 13 alcohols and polyols, 7 benzene derivatives, and 3 distinct polyamines (Fig. 5B).

Terpenoids and flavonoids are the principal secondary metabolites in *P. notoginseng* and exhibit extensive pharmacological activities [35]. MeJA treatment significantly altered the expression of 19 terpenoid compounds. We selected a subset of significantly altered terpenoid compounds for visualization and analysis. Following MeJA treatment, Notoginsenoside R_1_ exhibited a significant upward trend across all three time points compared with the control group. Notoginsenoside Ft_1_ showed a transient increase at 3 h posttreatment before stabilization, whereas Notoginsenoside Fe displayed minimal changes initially but demonstrated an accumulative trend after 24 h. Ginsenoside F_2_ increased initially at 3 h, stabilized thereafter, and increased again at 24 h. In contrast, Notoginsenoside R_2_ expression peaked at 3 h posttreatment and subsequently declined (Fig. 5C). Six flavonoid subclasses were found to be differentially expressed: chalcones, dihydrochalcones, anthocyanins, flavones, flavonols, flavan-3-ols, flavanones, and isoflavones. All were significantly upregulated in MeJA-treated samples, with anthocyanins and flavan-3-ols being less abundant than other subclasses (Fig. 5D).

Differential abundance analysis identified 382 significantly altered compounds (*P* < 0.05) between the MeJA_3h and control_3h groups (356 upregulated and 26 downregulated), 368 between the MeJA_6h and control_6h groups (344 upregulated and 24 downregulated), and 285 between the MeJA_6h and control_6h groups (213 upregulated and 72 downregulated). Compared with the controls, MeJA treatment significantly elevated Notoginsenoside R_1_, Ginsenoside Rf, (20S)-Ginsenoside Rh_2_, Ginsenoside Rg_1_, and Ginsenoside Re at 3 h. By 6 h, Ginsenoside Rg_1_, 5,6-Dehydroginsenoside Rd, Notoginsenoside R_2_, and Ginsenoside Rf decreased, whereas 20(R)-Ginsenoside Rh_1_ and (20S)-Ginsenoside Rh_2_ increased significantly. At 24 h, notoginsenosides R_1_, F_2_, and (20S)-Ginsenoside Rh_2_ had accumulated substantially (Fig. 5E–G). KEGG enrichment analysis revealed 51 metabolic pathways, including secondary metabolite biosynthesis, amino acid metabolism, and plant hormone signal transduction (Fig. 5H–J). These results indicated that MeJA elicited distinct metabolite accumulation patterns in *P. notoginseng* leaves, likely reflecting a coordinated regulatory response.

### Analysis of the correlation between *MYB* genes, saponin synthesis genes, and related metabolites

**Figure 6:**
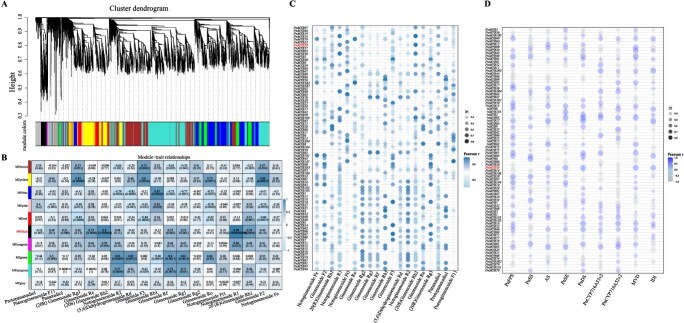
Co-expression module analysis of *PnMYB* genes under MeJA treatment. (A, B) Hierarchical clustering dendrogram of co-expression modules identified by WGCNA analysis, where each branch in the tree represents a gene. (C, D) Bubble plot of Pearson correlation coefficients between *PnMYB* and (C) 19 Triterpenoid saponin metabolites and (D) key triterpenoid biosynthesis genes.

Through weighted gene co-expression network analysis (WGCNA) of *PnMYBs*, 8001 genes were clustered into 10 modules after removing outliers. Based on the saponin metabolite trends under MeJA treatment (Fig. 6A), we selected 20 saponins that showed significant changes (ANOVA, *P* < 0.05) for the correlation analysis. The black (4090 genes) and green (2462 genes) modules were strongly correlated with multiple metabolites. The black module was positively correlated with the notoginsenosides R_1_, (20S)-ginsenoside Rh_2_, 20(R)-ginsenoside Rh_1_, ginsenoside Re, panaxadiol, and notoginsenoside Ft_1_. The green module positively correlated with notoginsenosides R_2_, 5,6-dehydroginsenoside Rd, ginsenoside Rf, ginsenoside Rg_1_, and ginsenoside Ro (Fig. 6B). A total of 17 highly connected hub genes were identified in the black module. Integrated hub gene screening and intramodular network analysis identified *PnMYB38* as a core transcriptional regulator (Fig. S3).

Spearman’s correlation analysis of the regulatory network among MYB TFs in *P. notoginseng*, 20 saponin metabolites, and 9 saponin biosynthesis genes. Several *MYB* genes (*PnMYB14, PnMYB16, PnMYB38*, *PnMYB70*, *PnMYB82*, *PnMYB89*, *PnMYB101*, *PnMYB103*, and *PnMYB106)* were significantly positively correlations with 4–10 saponin. Other correlated genes included *PnMYB1*, *PnMYB8, PnMYB20, PnMYB36, PnMYB46, PnMYB57, PnMYB69, PnMYB77, PnMYB78, PnMYB79, PnMYB81, PnMYB93, PnMYB98*, and *PnMYB3R2* (Fig. 6C). These findings indicated that MYB may have a regulatory function in saponin biosynthesis.

Notably, *PnMYB36*, *PnMYB38*, *PnMYB46*, *PnMYB57*, *PnMYB83*, *PnMYB94*, *PnMYB99*, and *PnMYB103* correlated strongly with *PnSS*, *PnSE*, *PnFPS*, *PnDS*, and *β-AS*. *PnMYB6*, *PnMYB13*, *PnMYB16*, *PnMYB30*, *PnMYB40*, *PnMYB81*, *PnMYB91*, *PnMYB95*, and *PnMYB3R3* were correlated with the other four saponin biosynthesis genes, whereas *PnMYB38, PnMYB46, PnMYB83, PnMYB99,* and *PnMYB103* were significantly correlated with the majority of saponin biosynthesis genes (Fig. 6D). Some *MYB* genes showed no significant correlation, implying roles in other processes (e.g. stress response, hormone signalling, or development) or the indirect regulation of saponin synthesis.

### *PnMYB38* binds to the promoters of both *PnSE* and *PnDS* and activates their expression

**Figure 7:**
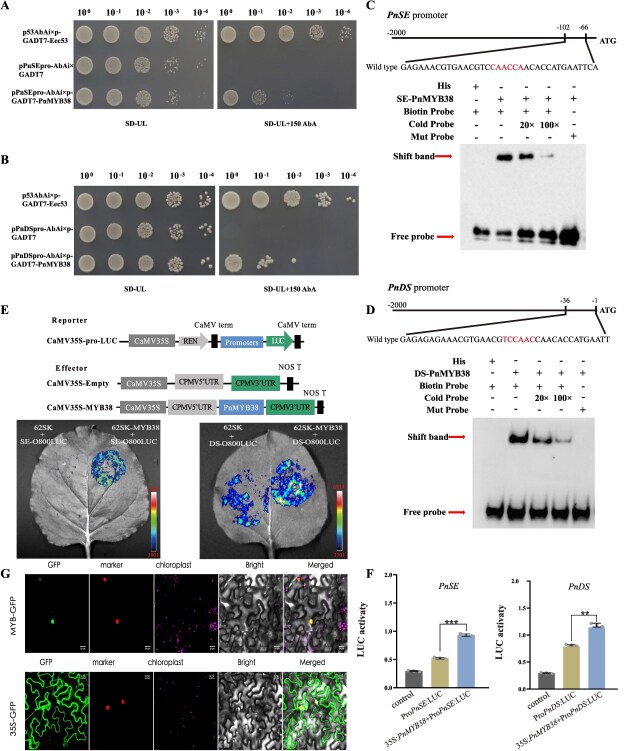
*PnMYB38* regulates the expression of *PnSE* and *PnDS* under MeJA treatment. (A, B) Y1H assay to verify interactions of *PnMYB38* with promoters *PnSE* and *PnDS*. (C, D) EMSA analysis demonstrating the direct binding of *PnMYB38* with promoters *PnSE* and *PnDS* promoter regions. (E) Effects of *PnMYB38* on the promoter activity of *PnSE* and *PnDS* as demonstrated by luciferase reporter assay. *PnMYB38* significantly affected the promoter activity of *PnSE* and *PnDS*. (F) Quantitative analysis of luminescence intensity. Three biological replicates were performed. The *P*-values were evaluated using Student’s *t*-test. Stars indicate the level of significance, ^*^0.01 < *P* < 0.05, and ^**^P < 0.01. (G) Subcellular localization of the *PnMYB38* protein. *PnMYB38* fused in-frame with green fluorescent protein (GFP) was transiently expressed in tobacco leaf cells. NLS-mkate is the marker of nucleus. Scale bars are 20 μm. Bright is image under normal field of view. Merge is overlay of the GFP, NLS-mkate, chloroplast, and bright images.

Three experimental approaches were employed to validate the transcriptional regulation of *PnMYB38* on saponin biosynthesis genes (*PnDS* and *PnSE*), three experimental approaches were employed. Yeast one-hybrid assays demonstrated that *PnMYB38* specifically binds to both the *PnSE* and *PnDS* promoters. The minimum inhibitory concentration of AbA was 150 ng/ml for the *PnSE* bait strain and 100 ng/ml for the *PnDS* bait strain (Figs S4 and S5). Robust growth was observed in yeast cells co-expressing *PnMYB38* and each promoter on SD/-Leu-Ura medium supplemented with the corresponding AbA concentration, whereas no growth was observed in the negative controls. Specific protein-DNA interactions were further confirmed through serial dilution spot assays, highlighting the DNA-binding specificity of *PnMYB38* (Fig. 7A and B). Electrophoretic mobility shift assays (EMSA) using prokaryotically expressed *PnMYB38* confirmed direct and specific binding to MBS motifs in the *PnDS* (TAACTG at −1176 bp) and *PnSE* (CAACCA at −1902 bp) promoters. Binding was abolished by cold-probe competition and mutation of the probe sequences (Fig. 7C and D). Dual-luciferase (LUC) reporter assays were performed using the tobacco transient expression system. The results showed that the relative LUC activity in leaves co-transformed with 35S:*PnMYB38* + Pro*PnDS*: LUC or 35S:*PnMYB38* + Pro*PnSE*: LUC was significantly increased compared to that in the controls (Fig. 7E and F; Table S11). These findings demonstrated that *PnMYB38* binds to and activates the promoters of *PnDS* and *PnSE*.

### Subcellular localization of *PnMYB38* protein

The *PnMYB38* coding sequence (CDS) (without a stop codon) was inserted into the pCAMBIA1300 vector and fused to green fluorescent protein (GFP)under the control of the CaMV35S promoter. The recombinant plasmid was transformed into Agrobacterium GV3101 and expressed in Nicotiana benthamiana leaves for 2 d postinfiltration. The *PnMYB38*-GFP fusion protein was observed in the nucleus and co-localized with the NLS-mKate2 red fluorescent signal. This localization aligned with earlier predictions, demonstrating that *PnMYB38* resides in the nucleus and participates in the transcriptional regulation of genes (Fig. 7G).

## Discussion

We identified 110 *PnMYB* genes from the *P. notoginseng* genome and compared them with the R2R3-type *MYB* genes of the model plants *A. thaliana* (123) [36], *P. ginseng* (117) [37], and *Panax quinquefolius* (159) [38]. *P. notoginseng* contains a relatively low number of *MYB* TFs. The smaller *MYB* family in *P. notoginseng* compared to its relatives (*P. ginseng* and *P. quinquefolius*) may be due to a lower rate of recent tandem duplication, fewer retained segmental duplicates, or more extensive gene loss after ancient polyploidy [34].

Further analysis of the *PnMYB* family showed significant differences in the length, molecular weight, and predicted isoelectric points of proteins encoded by different genes, which is consistent with previous studies. To explore the evolutionary relationships among genes, we constructed a phylogenetic tree of *A. thaliana* (123) and *P. notoginseng MYB* (110) family members and analyzed the characteristics and conservation of amino acid motifs in each subgroup. Groups 2, 14, 16, 17, and 19 showed amino acid sequence similarity to *Arabidopsis* R2R3-*MYB* gene family subgroups 9, 20, 23, 22, and 25, respectively, as proposed in previous studies [32]. The aa motif of group 13 closely resembled the reported WxPRL motif of *PnMYB78*. *PnMYB78* binds to the *PnFPS* and *PnSS* promoters to regulate the synthesis of saponins in *P. notoginseng* [39]. The *AtMYB4* (At4g38620) protein was found to function as both a transcriptional activator and repressor, and the aa motif of its subgroup was similar to that of Group 14 in our phylogenetic tree [32]. Additionally, they found that the expression of *AtMYB30* (AT3G28910) was closely related to cell death in hypersensitive responses triggered by pathogen attack or elicitor treatment and that the aa motif of its subgroup was similar to that of Group 8 in our phylogenetic tree. Members of subgroup 15, including *Arabidopsis* GL1 (AT5G40330.1), share a conserved 19-aa motif at the C-terminus. In *Arabidopsis*, GL1 is essential for initiating trichome differentiation, and the aa motif of subgroup 15 is similar to that of the branch containing *AtMYB61* [31]. *AtMYB74* and *AtMYB102* both in subgroup 11 and upregulated under drought conditions, exhibited motifs similar to those in Group 4 [40]. The functional diversity of the R2R3-*MYB* family is influenced by a conserved C-terminal motif outside the *MYB* domain, which may help to identify functional domains [32]. Thus, motif conservation across subgroups in *P. notoginseng* phylogenetic tree can aid in predicting the function of *MYB*.

A total of 103 *MYB* genes were mapped onto chromosomes in the chromosomal localization analysis, while the remaining seven genes could not be assigned to any chromosome. The *PnMYB* members of each subgroup showed a high degree of conservation in exon-intron number and length, with the number and proportion of introns and exons largely consistent with previous studies. Conserved motifs 1, 2, 3, and 4 were universally present in each *PnMYB* member, which is consistent with the conserved motifs 1, 2, and 3 identified in the *MYB* gene family of *P. quinquefolius* [38]. The amino acid composition of all motifs was highly similar to that of the previously characterized motifs of the *P. notoginseng MYB* gene family, differing only in numbering. Most cis-acting elements were associated with hormone responses and abiotic and biotic stresses, with light-responsive elements being the most common. This pattern is similar to the characteristics of cis-acting elements in the *MYB* family of *P. quinquefolius* [38]. Specific hormone-responsive cis-acting elements such as MeJA-responsive elements may play a role in stress responses and secondary metabolism. In barley, most *HvMYB* genes contain ABA-responsive, light-responsive, and MeJA-responsive elements [41]. Collinearity analysis identified 28 segmental duplication events and four tandem duplication events, indicating that the expansion of the *MYB* gene family in *P. notoginseng* was likely driven by segmental duplication events, a common evolutionary mechanism in plants. These findings were consistent with those of previous studies on *P. ginseng*, *P. quinquefolius*, and *P. notoginseng*. The diversity and evolutionary conservation of the *MYB* family underscore its functional importance in *P. notoginseng*.

*MYB* TFs are established regulators of flavonoid and terpenoid biosynthesis in plants [42–46], and MeJA has been proven to regulates triterpenoids and flavonoids biosynthesis in medicinal plants [47–51]. Studies have reported that MeJA induces the expression of *MYB* genes or other TFs that participate in the regulation of various aspects of plant growth and development. For instance, *PnMYB2* modulates JA and hypersensitivity pathways in the defense against *Fusarium solani* [52]. *PgMYB2* positively regulates dammarenediol synthase to promote ginsenoside biosynthesis in *P. ginseng* [26]. *AaMYB108* interacts with *AaGSW1* to promote artemisinin biosynthesis under light and JA [53]. Short-term MeJA treatment is the preferred strategy to enhance saponin biosynthesis and accumulation in *P. notoginseng* cultures and bioreactors. Transcriptome sequencing of *P. quinquefolius* seedlings treated with MeJA revealed that *PqMYBs* might play a regulatory role in the flavonoid pathway [38]. *AP2*/*ERF* TFs are upregulated in MeJA-treated *P. notoginseng* roots [54], and *GurMYB04* is induced in *Glycyrrhiza*, promoting flavonoid biosynthesis [55]. Analysis of MeJA-treated *P. ginseng* adventitious roots using WGCNA confirmed correlations between *MYB* TFs and ginsenoside biosynthesis [56].

Several *MYB* genes correlated strongly with key enzyme genes involved in saponin biosynthesis (e.g. *PnSS, PnSE, PnFPS, PnDS, Pnβ-AS, PnCYP, PnMVD, PnIDI*), supporting their involvement in saponin pathway regulation. The lack of correlation for some *MYB* genes may indicate indirect roles or functions in other processes (e.g. stress, hormone signaling, or development), consistent with multifunctional *MYB* characteristics [57, 58]. These findings agree with prior reports: MeJA activates JA signalling pathway, increased PPD-type saponins correlate with high expression of *FPS*, *SS*, *SE*, *DS*, and *UGTs*, as well as the low expression of *CYP716A53v2* and *β-AS* in *P. notoginseng* leaves [25]. *MsMYB* binds to the cis-regulatory element of *MsGPPS. LSU* to inhibit monoterpene biosynthesis [59]. *CiMYB42* is an important transcriptional activator involved in limonoid biosynthesis that regulates *CiOSC* expression by binding to the TTGTTG sequence (type II *MYB* core) [60]. MeJA promotes saponin accumulation and defense response in *P. notoginseng* via multiple TFs with divergent functions. For example, *PnMYB2* and *PnWRKY9* are involved in JA-mediated defense responses [52, 61], while *AP2*/*ERF* members correlate with saponin biosynthetic genes but lack confirmed direct regulation [54]. The metabolic branch regulator transducing MeJA signals to saponin biosynthetic genes remained unclear.

In the present study, MeJA significantly altered the expression of 84 *PnMYB* genes, with dynamic changes at 3, 6, and 24 h, indicating their key roles in the MeJA response. RT-qPCR validated the expression trends of the 10 selected *PnMYB* genes, corroborating the reliability of RNA-seq. Metabolomic analysis showed that MeJA increased the terpenoid and flavonoid levels in *P. notoginseng* leaves, suggesting the activation of relevant pathways. This trend was also observed in a study in which MeJA significantly promoted the accumulation of ginsenosides Rb_1_, Rc, Rb_2_, Rb_3_, and notoginsenoside Fa and Fe in *P. notoginseng* leaves [62].

This study identified *PnMYB38* as a key regulator, it directly binds to and activates promoters of key saponin biosynthetic genes *PnDS* and *PnSE*, acting as a metabolic pathway-specific transcriptional switch converting MeJA signals to saponin (especially dammarane-type) biosynthesis. WGCNA indicated association between *MYB* genes and saponin metabolites. The MEblack and MEgreen modules positively correlated with multiple saponins, indicating their regulatory roles. The black module exhibited the strongest correlation with saponin accumulation, where *PnMYB38* emerged as a core hub gene. This positions *PnMYB38* as a potential key regulator in the saponin biosynthetic pathway. Furthermore, among numerous MeJA-responsive DEGs, *PnMYB38* uniquely combines hormonal responsiveness with transcriptional control over saponin biosynthesis, thereby justifying its selection for subsequent functional characterization.

Transcriptional activation experiments confirmed that *PnMYB38* can directly bind to the promoter of *PnDS*, a key gene in dammarane-type saponin biosynthesis, and activate its expression. Targeted metabolomics analysis further revealed that, following MeJA treatment and upregulation of *PnMYB38* expression, the accumulation of notoginsenoside R_1_, a representative dammarane-type saponin, significantly increased at three time points (3, 6, and 24 h). These results collectively suggest that *PnMYB38* may promote the biosynthesis of dammarane-type saponins, particularly notoginsenoside R_1_, in *P. notoginseng* leaves by positively regulating *PnDS* transcription.

Functional assays confirmed the regulatory role of *PnMYB38* in saponin biosynthesis. Y1H, EMSA, and LUC assays demonstrated that *PnMYB38* directly binds to *PnDS* and *PnSE* promoters and activates transcription. This elucidates the mechanism by which MeJA-induced *PnMYB38* expression drives saponin biosynthesis via the direct activation of biosynthetic genes. Previous studies support *MYB*-mediated regulation of saponin biosynthesis that the *PnMYB61* TF might activate the expression of *PnSE2* [62]. *MYB*-mediated regulation of *SE* expression enhances dammarane-type triterpenoid saponin biosynthesis. The transient overexpression of *ZjWRKY18* upregulated key triterpenoid biosynthetic pathway genes, including *AACT*, *HMGR*, *FPS*, *SQS*, *SQE*, *OSC*, and *CYP450*, resulting in increased triterpenoid accumulation in jujube fruit. *ZjWRKY18* may interact with the promoters of triterpenoid biosynthesis-related genes in plants [63].

## Conclusion

**Figure 8:**
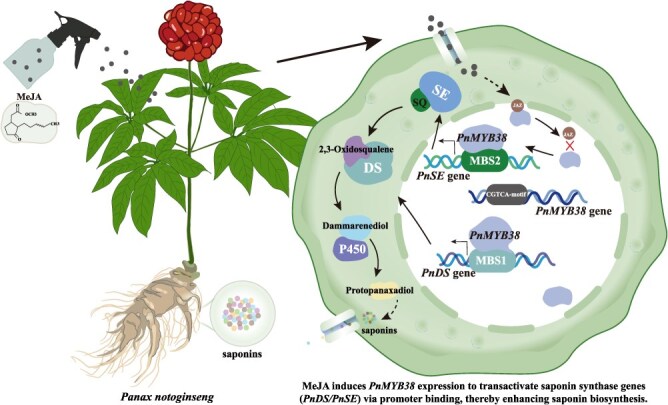
A model for the role of *PnMYB38* in MeJA-induced saponin biosynthesis.

This study provides a comprehensive analysis of the *MYB* TF family in *P. notoginseng* and elucidates its role in MeJA-induced metabolic regulation and saponin biosynthesis. We identified 110 *PnMYB* genes and classified them into 19 subgroups using phylogenetic analysis. Certain subgroups exhibited amino acid motifs similar to those of *Arabidopsis MYB* genes, suggesting that these genes have retained their functions throughout evolution, although species-specific divergence was also evident. Gene structure and motif analyses showed evolutionary diversification, while retaining key conserved motifs that are likely to be involved in critical biological processes. Cis-element analysis revealed abundant stress- and hormone-responsive elements, particularly MeJA-responsive elements, highlighting the central role of *MYB* family in MeJA-mediated metabolic regulation. Chromosomal mapping and synteny analysis indicated that family expansion was primarily driven by segmental duplication, emphasizing the evolutionary role of gene duplication. MeJA treatment significantly altered the expression patterns of 84 *MYB* genes, with dynamic changes observed at 3, 6, and 24 h, underscoring their importance in the MeJA response. Metabolomic analysis confirmed increased terpenoid and flavonoid levels in *P. notoginseng* leaves, indicating that MeJA activates the relevant metabolic pathways. WGCNA and correlation analyses revealed co-expression networks linking *MYB* genes, saponin metabolites, and biosynthetic enzymes (e.g. *PnSE* and *PnDS*). *PnMYB38* functioned as a core regulator of saponin biosynthesis, with functional validation demonstrating its binding and activation of *PnSE* and *PnDS* promoters, thereby establishing its pivotal role in MeJA-induced saponin biosynthesis. Sequence analysis revealed that the promoter region of *PnMYB38* contains the typical jasmonate-responsive element CGTCA/TGACG, which serves as a recognition site for downstream TFs, such as the *MYC2* complex, in the JA signaling pathway [64]. In addition, according to the classic model of JA signaling, JAZ proteins are degraded via the SCF(COI1) pathway in the presence of JA-Ile, thereby relieving their inhibitory effects on downstream TFs [53, 65, 66]. This mechanism may be involved in the regulation of *PnMYB38* expression. This study primarily focuses on the downstream regulatory functions of *PnMYB38*, whereas its specific upstream interaction network requires further validation (Fig. 8).

In summary, this study elucidates the regulatory network of secondary metabolism in *P. notoginseng* and provides foundational insights for its genetic engineering. In the future, researchers will employ the CRISPR/Cas9 gene-editing technology to further elucidate the precise regulatory mechanisms by which *MYB* genes control saponin biosynthesis in *P. notoginseng*.

## Materials and methods

### Plant material

Three-year-old *P. notoginseng* plants were used in the present study. The plants were obtained from the *P. notoginseng* planting base in Wenshan County, Yunnan Province, China (N 23°23′20.26″, E 104°13′49.49″; altitude: 1260 m) and grown in the greenhouse of Kunming University of Science and Technology (N 24°51′27″, E 102°51′12″; altitude: 1913.67 m). Following a 2-week acclimatization period (20 ± 2°C, 70% humidity, 16-h light/8-h dark cycle), plants were foliar-sprayed with 100 μmol/l MeJA containing 0.005% (v/v) Silwet L-77 for 10 min. Control plants received an equivalent volume of 0.005% (v/v) Silwet L-77 solution without MeJA. Leaves were collected before (0 h) and after MeJA treatment (3, 6, and 24 h post treatment). After quick freezing with liquid nitrogen, the leaves were stored at −80°C until further use. Transcriptome sequencing, metabolomic sequencing, and qRT-PCR were performed.

### Identification of the *MYB* gene family in *P. notoginseng*

The Hidden Markov Model (HMM) file for the MYB-binding domain (PF00249) was downloaded from Pfam (http://pfam.xfam.org/) [67–70]. HMMER software v3.3.2 was used to search the *P. notoginseng* acid sequence sequences for matches, with a preliminary E-value cut-off of <1e-3. Candidate genes were subjected to multiple sequence alignment using ClustalW v2.1, and a species-specific HMM was constructed from the alignment results. This model was used for the subsequent HMM searches, with an E-value cutoff of 1e-5 [67–70]. In total, 123 *A. thaliana* MYB protein sequences were downloaded from the TAIR database (https://www.arabidopsis.org/) on 19 January 2024. These sequences were used as queries for BLAST against the *P. notoginseng* genome data (NCBI BioProject ID PRJNA658419) using TBtools software v2.069 (South China Agricultural University, Chen Chengjie, China), with an E-value cutoff of 1e-5. The intersection of candidates identified by both the HMM and BLAST searches was considered the initial set of MYB family members. Final validation was performed using online tools, including SMART (http://smart.embl.de/), InterPro (https://www.ebi.ac.uk/interpro/), and NCBI-CDD (https://www.ncbi.nlm.nih.gov/cdd/) to screen and confirm whether the *PnMYB* protein sequence contains a complete SANT/myb DNA-binding domain (screening results are in Table S3). The ExPASy website (https://web.expasy.org/compute_pi/) was used to analyze the physicochemical properties of the MYB proteins, including amino acid number, relative molecular weight, theoretical isoelectric point, instability index, aliphatic index, and hydrophilicity. Subcellular localization was predicted using the WOLF PSORT tool (https://www.genscript.com/wolf-psort.html).

### Phylogenetic tree construction, chromosomal localization, and collinearity analyses of *MYB* genes

A phylogenetic tree including *MYB* family members from *P. notoginseng* (110 sequences) and *A. thaliana* (124 sequences) was constructed using the Maximum Likelihood (ML) method in IQ-TREE v2.2.6. The best-fit substitution model identified for tree construction was JTTDCMut+F + R6, which was chosen according to the BIC. The tree was visualized using Evolview (https://evolgenius.info//evolview-v2/). *PnMYB* genes were classified into different subgroups (R2R3- and *MYB*-related) based on the manual identification of *MYB* domain repeats and were subsequently named according to their genomic coordinates. Chromosomal locations and gene density were visualized using the ‘Visualize Gene Location’ and ‘Gene Density’ functions in TBtools.

Synteny refers to the conserved order of genes along a chromosome. Many genes in a species exist in multiple copies rather than single copies, leading to gene duplication events. Synteny analysis was performed using the MCScanX toolkit implemented in TBTools. Genome and annotation (GFF3) files of *P. notoginseng* were used as inputs. The results were visualized with the ‘Dual Synteny Plot for MCScanX’ function in TBtools.

### Characterization of conserved motifs, gene structure, and cis-regulatory elements in the *PnMYB* family

Conserved protein motifs were identified using the MEME Suite (http://meme-suite.org/), with the number of motifs set to 10 and other parameters kept at default values. Gene structure diagrams (exon-intron organization) were generated using TB tools. The 2000-bp promoter regions of the translation start site of each *PnMYB* gene were extracted. Putative cis-regulatory elements were identified using the PlantCARE database (https://bioinformatics.psb.ugent.be/webtools/plantcare/html/). The results were visualized using R v 4.1.3.

### RNA-seq and qRT-PCR analysis

Sequencing was perform on an Illumina HiSeq™ 4000 platform (San Diego, CA, USA), generating 150-bp paired-end reads. Raw reads were quality-filtered using Fastp v0.23.4 to obtain clean reads. The *P. notoginseng* genome (PRJNA658419) was used as the reference. Clean reads were aligned to the reference genome using Hisat2 v2.2.1, achieving an average mapping rate of 79.25%. Mapped reads were processed using Samtools v1.17 and Stringtie v2.2.1, to calculate transcript abundance and generate a normalized expression matrix. Gene expression levels are expressed as log10 (TPM + 1).

Total RNA was extracted from the control and treated samples. Reverse transcription was performed using the SynScript III RT SuperMix for qPCR (Qingke). The resulting cDNA products were diluted 1:3 and used as templates for qPCR amplification using the Qingke ArtiCanCEO SYBR qPCR Mix. The reaction mixture contained 10 μl of ArtiCanCEO SYBR qPCR Mix, 0.8 μM of each forward and reverse primer, and 1 μl of cDNA template, and sterile water was added to bring the total volume to 20 μl. PCR was performed under the following thermal cycling conditions: 95*°*C for 5 min, followed by 40 cycles of 95°C for 15 s, 60°C for 20 s, and 72°C for 20 s. The actin gene was used as an internal reference. For the MeJA induction experiment, gene expression levels were normalized to those of the untreated control (0 h). Relative expression was calculated using 2^-ΔΔCt^ method. The primer sequences used for cloning are listed in Table S12.

### Metabolome analysis

Frozen tissues (100 mg) were ground in liquid nitrogen and transferred into tubes. 500 μl of 80% aqueous methanol was added, followed by vortexing and incubation on ice for 5 min. The sample was centrifuged at 15 000 × *g* at 4°C for 20 min. The supernatant was collected, diluted with MS-grade water to a final methanol concentration of 53%, and centrifuged under the same conditions. The final supernatant was injected into the LC–MS system for analysis.

The analysis was performed using an LC-ESI-MS/MS system (UPLC: Shim-pack UFLC SHIMADZU CBM30A; MS: Applied Biosystems 6500 QTRAP). A 5-μl aliquot was injected onto an XSelect HSS T3 XP column (2.1 × 150 mm, 2.5 μm). The mobile phases were 0.1% formic acid in water (A) and 0.1% formic acid in acetonitrile (B). The gradient elution programs were 0–15 min, 2%–100% B; 15–17 min, 100% B; and 17.1–20 min, 2% B. The flow rate was 0.4 ml/min. The ESI source parameters were as follows: temperature, 550°C; ion spray voltage, 5500 V; and curtain gas, 35 psi. The MRM mode was used for detection with optimized declustering potentials and collision energies for each transition. The mass scan range was *m*/*z* 50–1000.

Metabolites were annotated using the KEGG (https://www.genome.jp/kegg/pathway.html), HMDB (https://hmdb.ca/metabolites), and LIPIDMaps databases (http://www.lipidmaps.org/). Multivariate statistical analyses, including PCA and partial least squares discriminant analysis, were performed using metaX software. Metabolites with a variable importance in projection (VIP) score > 1, *P*-value < 0.05 (Student’s *t*-test), and fold change (FC) ≥ 2 or ≤−2 were considered significantly differential. Volcano plots, heat maps (using *z*-scores), correlation analysis (Pearson, visualized with corrplot), and KEGG pathway enrichment were generated using R v4.1.3 and related packages (ggplot2 and pheatmap). A pathway was considered significantly enriched if the *P*-value was <0.05.

### Correlation analysis of the transcriptome and metabolome

We employed weighted gene co-expression network analysis (WGCNA) to construct co-expression networks for *PnMYB* genes and to investigate their potential roles in saponin biosynthesis. The co-expression network was generated using the WGCNA package (v1.73) in R. Gene expression matrices from four time points were used as input for the weighted gene co-expression analysis. After filtering (genes expressed in ≥50% of samples, expression cutoff = 5, filtering method = median absolute deviation), a total of 6000 genes were retained for subsequent analysis. The optimal soft-thresholding power was selected to achieve a scale-free topology, and a hierarchical clustering tree was constructed using the following parameters: minimum module size = 30, cut height for modules merging = 0.25, and maximum block size = 6000. Spearman’s rank correlation coefficients were calculated between module eigengenes and the expression of key saponins biosynthesis enzyme genes (*PnSS, PnSE, PnFPS, PnDS*, *Pnβ-AS, PnCYP, PnMVD,* and *PnIDI*), as well as between module eigengenes and saponin content. The analytical methodology [71] was followed.

### Subcellular localization of *PnMYB38*

The coding sequence of *PnMYB38* was amplified and inserted into the pCAMBIA1300-35S-GFP vector to generate an N-terminal GFP fusion under the control of the CaMV 35S promoter (pCAMBIA1300-35S-*PnMYB38*-GFP). The plasmid was then transformed into *Agrobacterium tumefaciens* GV3101 via electroporation. Transformed agrobacteria were cultured in YEB medium with appropriate antibiotics at 30°C for 2 days, then subcultured and grown to an OD600 of ~0.6. The bacterial cells were harvested and resuspended in infiltration buffer (10 mM MgCl₂, 120 μM acetosyringone). The suspension was infiltrated into the abaxial side of tobacco leaves using a syringe. After incubation in the dark for 2 days, GFP fluorescence was observed using a laser scanning confocal microscope (Nikon C2-E). GFP was excited at 488 nm, the emission was detected at 510 nm, and chlorophyll autofluorescence was excited at 640 nm and detected at 675 nm. The primers used for PCR amplification are listed in Table S13.

### Dual-luciferase assay

The promoter sequences of *PnDS* and *PnSE* (Table S14) were cloned into the pGreenII 0800 vector to generate reporter constructs (pGreenII 0800-pro*PnDS*-LUC, pGreenII 0800-pro*PnSE*-LUC). The CDS of *PnMYB38* was cloned into pGreenII 62-SK to generate the effector construct (pGreenII 62-SK-*PnMYB38*). These constructs were transformed into *A. tumefaciens* GV3101. The bacterial suspension was infiltrated into the leaves of 4-week-old tobacco plants. After incubation in the dark for 24 h followed by 24–48 h under light, the leaf discs were harvested. Luciferase activity was measured using a dual-luciferase assay kit (Yeasen Biotech, Shanghai, China) and a Tanon-5200 Chemiluminescence Imaging System. Firefly luciferase (LUC) activity was normalized to renilla luciferase (REN) activity. The primers used are listed in Table S13.

### Yeast one-hybrid

The promoter fragments *PnSE* (683 bp) and *PnDS* (2000 bp) were cloned into the pAbAi vector to generate bait constructs, whereas the coding sequence of *PnMYB38* was inserted into pGADT7 to create the effector plasmid. Linearized bait plasmids were transformed into *S. cerevisiae* Y1H gold-competent cells using PEG/LiAc-mediated transformation, and positive clones were verified by PCR. The minimum inhibitory concentration of aureobasidin A (AbA) for each bait strain was determined using titration assays (0–1000 ng/ml). For the interaction analysis, bait strains were co-transformed with pGADT7-*PnMYB38* (experimental), empty pGADT7 (negative control), or pGADT7-Rec53 (positive control). Protein-DNA interactions were confirmed by growth on SD/-Leu-Ura plates supplemented with strain-specific AbA concentrations after 4–5 days at 30°C, with validation via serial dilution spot assays using normalized cell densities (OD_600_ = 1 to 10^−4^). The primers used to generate various clones are listed in Table S13.

### Electrophoretic mobility shift assay

The CDS of *PnMYB38* was cloned into the pET-28a (His-tag) vector for expression as a His-tagged fusion protein in *Escherichia coli* BL21(DE3). The recombinant protein was purified using Ni-NTA beads 6FF (Smart-Lifesciences) according to the manufacturer’s instructions. Biotin-labelled DNA probes corresponding to the putative binding sites in the target gene promoters were synthesized by Sangon Biotech. EMSA was performed using a commercial kit (Beyotime Biotechnology). Protein-DNA binding reactions were incubated, separated on nondenaturing polyacrylamide gels, and transferred to nitrocellulose membranes. Biotin-labelled DNA was detected using chemiluminescence. The primers used for cloning are listed in Table S13.

## Supplementary Material

Web_Material_uhag052

## Data Availability

The RNA-seq data have been deposited at the China National GeneBank (CNGBdb, https://db.cngb.org, Project number: PRJCA048040) and are publicly available as of the date of publication.
