# Supplementary material for: The transcription factor PnMYB38 orchestrates methyl jasmonate-induced saponin biosynthesis in Panax notoginseng
Source: Hortic Res. 2026 Feb 18;13(6):uhag052. doi: 10.1093/hr/uhag052 (PMC13241188; doi:10.1093/hr/uhag052)
Supplement: Web_Material_uhag052 [file web_material_uhag052.zip › Figure and Table informations.docx]

**Figure legends**

**Figure 1.** Gene structure, conserved motif, and conserved domain analysis of *PnMYB* genes. (A) *PnMYB* genes family phylogenetic tree and conserved Motif, Domains of the *PnMYB* genes family, Exon–intron regions of the *PnMYB* genes family. (B) The ten motifs of *PnMYB* genes in detail.

**Figure 2.** Analysis of *PnMYB* genes promoter elements of *Panax notoginseng*. (A) Distribution pattern of cis-acting elements in the *PnMYB* promoter region. (B) Promoter region cis-acting element quantity heat map. (C) Number of *PnMYB* genes in Growth and development, Biological and abiotic, Hormone response, and Other.

**Figure 3.** Chromosomal localization, phylogenetic relationship and the collinearity analysis within the genome of *PnMYBs*. (A) Chromosomal localization of *P. notoginseng* MYB genes. (B) a maximum likelihood phylogenetic tree of *PnMYB* genes and *AtMYB* genes. The phylogenetic tree was constructed using the Maximum Likelihood (ML) method in IQ-TREE v2.2.6. Use the online site Evolview for evolutionary tree beautification. And the nineteen major groups are marked with different color backgrounds. (C)The collinearity analysis within the genome of *PnMYB*.

**Figure 4.** Expression analysis of *PnMYB* and saponin biosynthetic genes in response to MeJA treatment. (A) Heatmap of the differentially expressed 110 *PnMYB* genes family during MeJA treatment. (B) Heatmap analysis of biosynthetic gene expression dynamics in the saponin pathway of *Panax notoginseng*. (C, D) RT-qPCR validation of MYB TF and key triterpenoid biosynthesis gene expression during MeJA treatment.

**Figure 5.** Evaluation of the influence of protoplasting genes on cell clustering. (A) PCA of Metabolites: Treatment vs. Control at 3 time points. (B) Radar chart of metabolite proportions between treatment and control groups at three time points**.** (C, D) Differential analysis of terpenoids and flavonoids in treatment vs. control groups at three time points. (E-G) Volcano plots of DAMs between MeJA-treated and control groups at three time points. (H-J) KEGG enrichment plot of DAMs between MeJA-treated and control groups at three time points.

**Figure 6.** Co-expression module analysis of *PnMYB* genes under MeJA treatment. (A, B) Hierarchical clustering dendrogram of co-expression modules identified by WGCNA analysis, where each branch in the tree represents a gene. (C, D) Bubble plot of Pearson correlation coefficients between *PnMYB* and (C) 19 Triterpenoid saponin metabolites, (D) Key triterpenoid biosynthesis genes

**Figure 7.** *PnMYB38* regulates the expression of *PnSE* and *PnDS* under MeJA treatment. (A, B) Y1H assay to verify interactions of *PnMYB38* with promoters *PnSE* and *PnDS*. (C, D) EMSA analysis demonstrating the direct binding of *PnMYB38* with Promoters *PnSE* and *PnDS* promoter regions. (E) Effects of *PnMYB38* on the promoter activity of *PnSE* and *PnDS* as demonstrated by luciferase reporter assay. *PnMYB38* significantly affected the promoter activity of *PnSE* and *PnDS*. (F) Quantitative analysis of luminescence intensity. Three biological replicates were performed. The p-values were evaluated using Student’s t-test. Stars indicate the level of significance, *0.01 < p < 0.05, and **p < 0.01. (G) Subcellular localization of the *PnMYB38* protein. *PnMYB38* fused in-frame with green fluorescent protein (GFP) was transiently expressed in tobacco leaf cells. NLS-mkate is the marker of nucleus. Scale bars are 20 μm. Bright is image under normal field of view. Merge is overlay of the GFP, NLS-mkate, chloroplast and bright images.

**Figure 8.** A model for the role of *PnMYB38* in MeJA-induced saponin biosynthesis. *PnMYB38* expression to transactivate saponin synthase genes (*PnDS/PnSE*) via promoter binding, thereby enhancing saponin biosynthesis.

**Supplementary Information**

**Figure S1.** PCA score plot of the transcriptome across time points in MeJA-treated *P. notoginseng* leaves. Set three biological replicates for each treatment.

**Figure S2.** Volcano plots visualizing DEGs in *P. notoginseng* leaves after MeJA treatment.

**Figure S3.** Hub gene interaction network in the WGCNA black module. Through WGCNA analysis of the black module, we identified 17 highly interconnected hub genes. Subsequent screening and intramodular network analysis revealed *PnMYB38* as the central transcriptional regulator.

**Figure S4.** Determination of minimal AbA inhibitory concentration for *PnSE* Yeast transformants. The p53-AbAi strain served as the positive control, exhibiting complete growth suppression at the standard AbA concentration of 200 ng/mL. As shown in the figure, robust colony growth was observed in the absence of AbA, while growth inhibition upon AbA addition confirmed proper system functionality. For the experimental pPnSEpro-AbAi strain, no colonies formed at a lower AbA concentration of 150 ng/mL, establishing this as the minimal inhibitory concentration for subsequent yeast one-to-one interaction assays.

**Figure S5.** Determination of minimal AbA inhibitory concentration for *PnDS* Yeast transformants. The p53-AbAi strain served as the positive control, exhibiting complete growth inhibition at the standard AbA concentration of 200 ng/mL. Robust colony growth was observed in the absence of AbA, while effective suppression upon AbA addition confirmed proper system functionality. For the experimental pDSpro-AbAi strain, complete growth inhibition occurred at a reduced AbA concentration of 100 ng/mL, establishing this as the minimal inhibitory concentration suitable for subsequent yeast one-to-one interaction assays.

**Table S1**. Identification of MYB gene family members via HMM and BLAST searches in *P. notoginseng*.

**Table S2**. Analysis of physical and chemical properties of *PnMYBs*.

**Table S3.** The domain of *PnMYBs*.

**Table S4.** The number of cis-acting elements of *PnMYB* genes

**Table S5.** *PnMYB* cis-acting elements distribution

**Table S6.** Gene mapping on chromosomes and MYB members of *P. notoginseng*.

**Table S7.** Phylogenetic tree grouping patterns.

**Table S8.** The gene replication mode of *PnMYBs.*

**Table S9.** List of all gene identified in *P. notoginseng*.

**Table S10.** List of all metabolites identified in *P. notoginseng*.

**Table S11.** Relative luciferase activity (LUC/REN ratio) data.

**Table S12.** The primers list used for qRT-PCR.

**Table S13.** Primer sequences for gene amplification, vector construction, Dual-luciferase report assays, Electrophoretic mobility shift assay, Yeast One-Hybrid Assays and Subcellular localization.

**Table S14.** The promoter of *PnSE*, *PnDS* sequence for Dual-luciferase report assays, Electrophoretic mobility shift assay and Yeast One-Hybrid Assays.
