# Supplementary material for: The transcription factor PnMYB38 orchestrates methyl jasmonate-induced saponin biosynthesis in Panax notoginseng
Source: Hortic Res. 2026 Feb 18;13(6):uhag052. doi: 10.1093/hr/uhag052 (PMC13241188; doi:10.1093/hr/uhag052)
Supplement: Web_Material_uhag052 [file web_material_uhag052.zip › Supplemental Figures.docx]

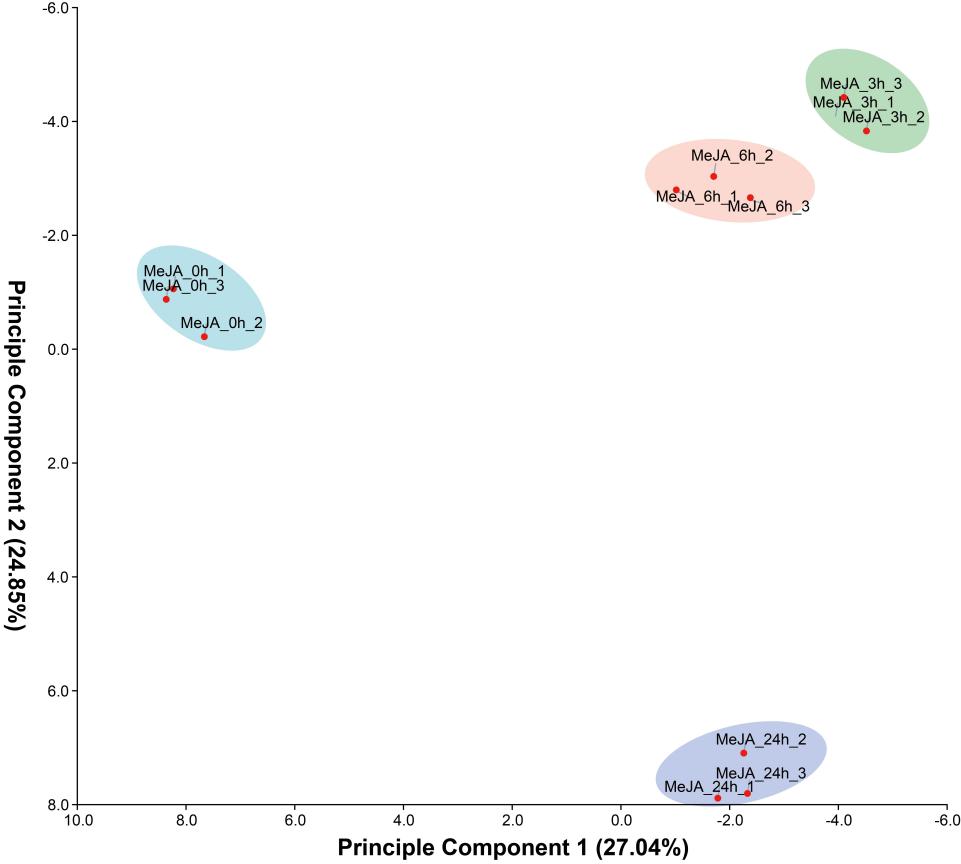


**Figure S1.** PCA score plot of the transcriptome across time points in MeJA-treated *P. notoginseng leaves.* Set three biological replicates for each treatment.


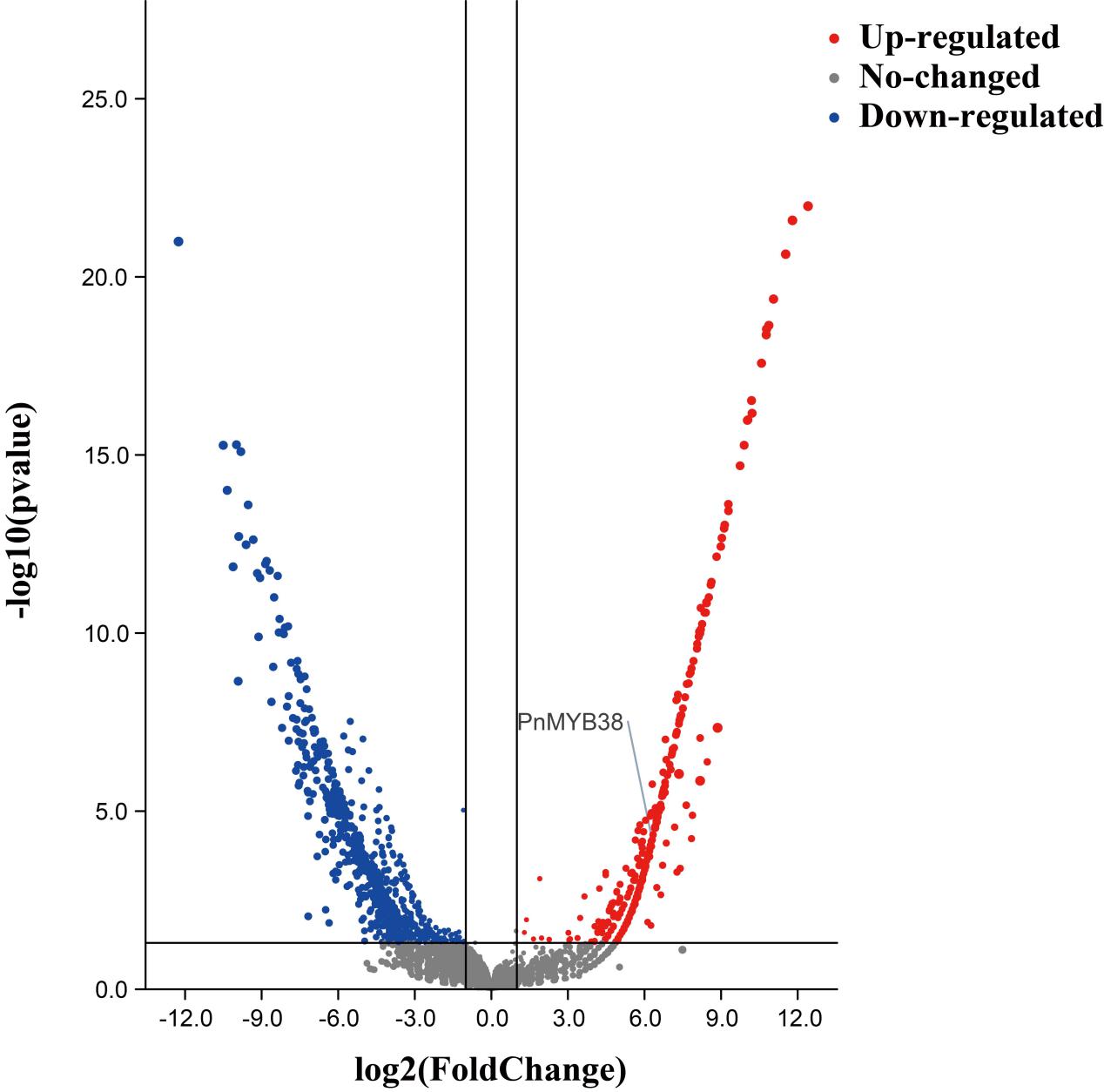


**Figure S2.** Volcano plots visualizing DEGs in *P. notoginseng* leaves after MeJA treatment.


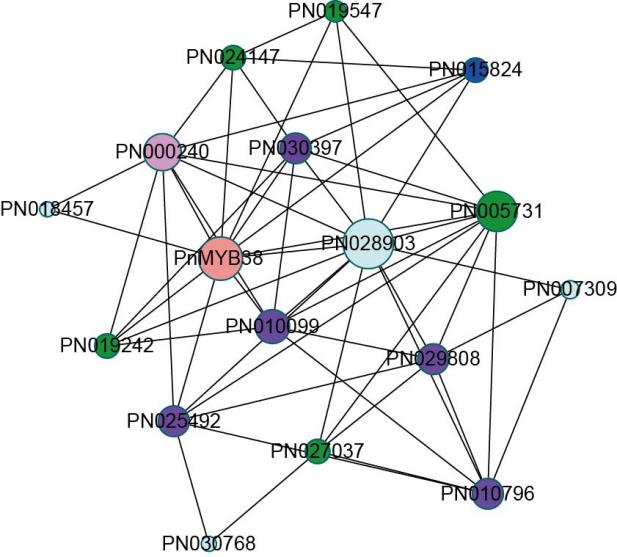


**Figure S3.** Hub gene interaction network in the WGCNA black module. Through WGCNA analysis of the black module, we identified 17 highly interconnected hub genes. Subsequent screening and intramodular network analysis revealed *PnMYB38* as the central transcriptional regulator.


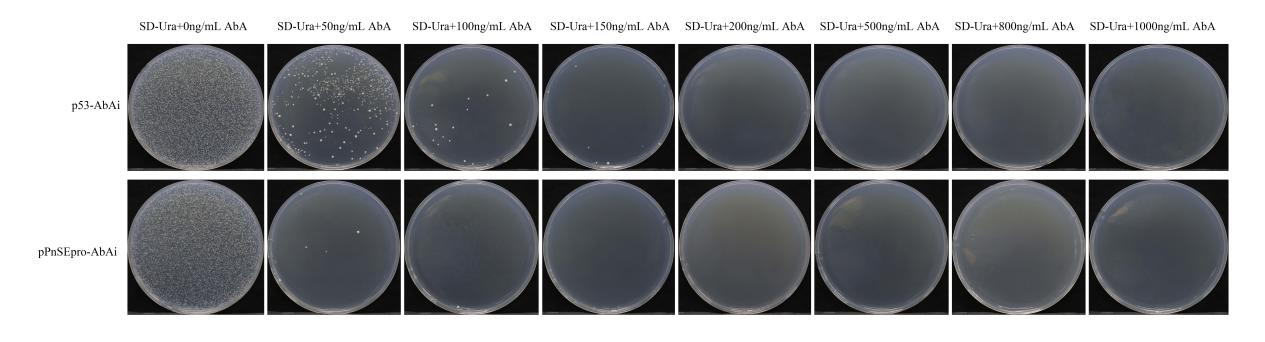


**Figure S4.** Determination of minimal AbA inhibitory concentration for *PnSE* Yeast transformants. The p53-AbAi strain served as the positive control, exhibiting complete growth suppression at the standard AbA concentration of 200 ng/mL. As shown in the figure, robust colony growth was observed in the absence of AbA, while growth inhibition upon AbA addition confirmed proper system functionality. For the experimental pPnSEpro-AbAi strain, no colonies formed at a lower AbA concentration of 150 ng/mL, establishing this as the minimal inhibitory concentration for subsequent yeast one-to-one interaction assays.


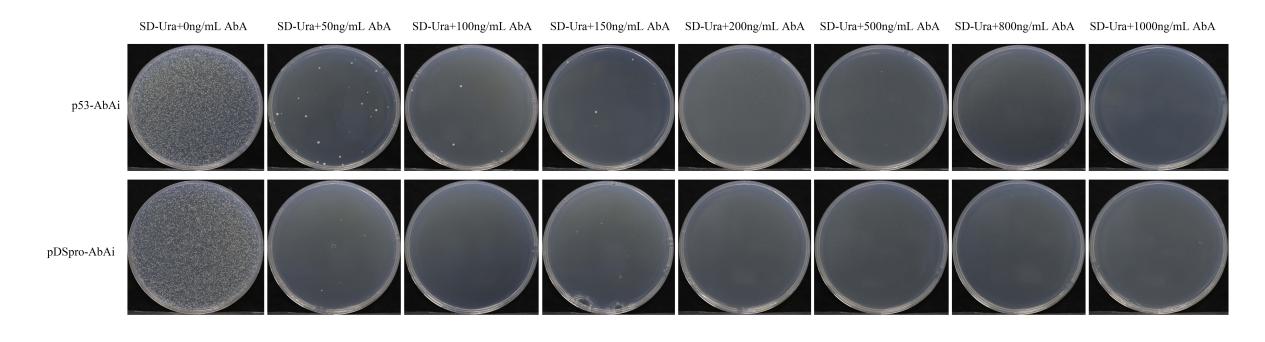


**Figure S5.** Determination of minimal AbA inhibitory concentration for *PnDS* Yeast transformants. The p53-AbAi strain served as the positive control, exhibiting complete growth inhibition at the standard AbA concentration of 200 ng/mL. Robust colony growth was observed in the absence of AbA, while effective suppression upon AbA addition confirmed proper system functionality. For the experimental pDSpro-AbAi strain, complete growth inhibition occurred at a reduced AbA concentration of 100 ng/mL, establishing this as the minimal inhibitory concentration suitable for subsequent yeast one-to-one interaction assays.
